# Hidden hysteresis – population dynamics can obscure gene network dynamics

**DOI:** 10.1186/1754-1611-7-16

**Published:** 2013-06-24

**Authors:** Phillip Poisson, Kaustubh D Bhalerao

**Affiliations:** 1 Kimberly-Clark Corporation, Neenah, WI, USA; 2 Department of Agricultural and Biological Engineering, University of Illinois at Urbana-Champaign, 1304 W Pennsylvania Ave, Urbana, IL 61801, USA

## Abstract

**Background:**

Positive feedback is a common motif in gene regulatory networks. It can be used in synthetic networks as an amplifier to increase the level of gene expression, as well as a nonlinear module to create bistable gene networks that display hysteresis in response to a given stimulus. Using a synthetic positive feedback-based tetracycline sensor in *E. coli*, we show that the population dynamics of a cell culture has a profound effect on the observed hysteretic response of a population of cells with this synthetic gene circuit.

**Results:**

The amount of observable hysteresis in a cell culture harboring the gene circuit depended on the initial concentration of cells within the culture. The magnitude of the hysteresis observed was inversely related to the dilution procedure used to inoculate the subcultures; the higher the dilution of the cell culture, lower was the observed hysteresis of that culture at steady state. Although the behavior of the gene circuit in individual cells did not change significantly in the different subcultures, the proportion of cells exhibiting high levels of steady-state gene expression did change.

Although the interrelated kinetics of gene expression and cell growth are unpredictable at first sight, we were able to resolve the surprising dilution-dependent hysteresis as a result of two interrelated phenomena - the stochastic switching between the ON and OFF phenotypes that led to the cumulative failure of the gene circuit over time, and the nonlinear, logistic growth of the cell in the batch culture.

**Conclusions:**

These findings reinforce the fact that population dynamics cannot be ignored in analyzing the dynamics of gene networks. Indeed population dynamics may play a significant role in the manifestation of bistability and hysteresis, and is an important consideration when designing synthetic gene circuits intended for long-term application.

## Introduction

Positive feedback is frequently seen as a regulatory mechanism in natural and synthetic gene networks [1-6]. Bistability and hysteresis are two salient functional features of positive feedback frequently exploited in synthetic gene networks [1,7-11]. A bistable response constrains gene expression to one of two discrete, steady-state levels, conventionally named high / low or on / off states. The choice of the expression state is generally governed by the initial conditions of the system, which includes any prior gene expression history. Consequently, bistable systems exhibit some degree of hysteresis, i.e. memory of prior induction levels. A number of groups have studied natural and synthetic positive feedback gene networks in bacterial and mammalian hosts and have produced experimentally validated mathematical models to predict hysteretic behavior in transcriptional gene regulation [1,8,12,13]. From the models, it becomes clear that although positive feedback can produce a bistable response in principle, bistability is not a guaranteed outcome of positive feedback. The appearance of hysteresis depends strongly on the relative production and degradation rates of the biomolecules responsible for expression regulation. A purely bistable response generally requires positively cooperative feedback, and an optimal balance of gene product synthesis and degradation [9,10,14,15].

Under exceptional circumstances bistability can also appear without the presence of a positively cooperative positive feedback within the gene circuit, as long as there is another mechanism that can introduce the requisite non-linearity. For instance, a non-cooperative positive feedback can introduce the appearance of bistability when gene expression dynamics are coupled with a non-linear modulation of growth rate [16], or in the presence of significant noise [17]. Unsurprisingly, mechanisms that induce hitherto unforeseen nonlinear or stochastic behaviors erode the predictability of artificial gene networks outside well-controlled experiments. This unpredictability is frequently referred to as context-dependent behavior in synthetic gene networks [18,19], and remains one of the foremost challenges in realizing the grand vision of synthetic biology, namely the construction of arbitrary genetic and biochemical networks from standardized component parts [20,21].

In addition to the two scenarios described above, (namely seeing hysteresis seen where we expect it, and seeing hysteresis where we do not expect it) we report a third, and (to the best of our knowledge) previously unreported scenario where we do not see hysteresis even though a biochemical model of gene expression predicts its presence. This work posits the influence of population dynamics in obscuring the appearance of hysteresis in gene networks. In this study we revisited the previously developed positive feedback based genetic signal amplifier [4] and examined it for hysteresis when the cell culture is propagated through batch cultures. We note that there exist bacterial growth theories which elucidate changes in gene expression as a function of growth rate in continuous, steady state, nutrient limited “balanced exponential” cultures [22,23], which control the culture in a state of constant exponential growth. Significantly, these theories describe protein synthesis rates solely as a function of cell growth rate, which in turn is dependent upon the nutrient state of the cell. In batch cultures, neither cell growth rates nor gene expression profiles remain constant. Thus balanced exponential models of growth are not directly applicable to changes in transcription patterns that occur as a result of achieving stationary state through nutrient depletion in batch cultures.

**Figure 1:**
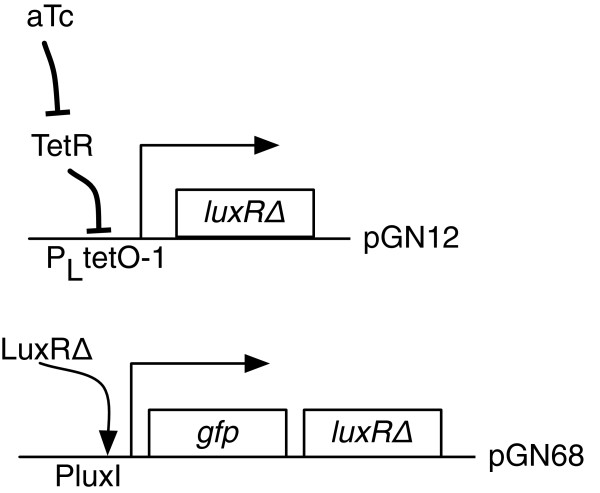
**The positive feedback gene network [**[4]**].** Cotransformation with plasmids pGN12 and pGN68 results in a positive feedback circuit. Inducer anhydrotetracycline (aTc) induces the copy of *luxRΔ* from pGN12. Activator LuxR *Δ* binds to its cognate promoter *PluxI* and induces another copy of *luxRΔ*, thereby constituting positive feedback.

A schematic of the gene circuit tested in this paper is shown in Figure 1. The positive feedback amplifier was originally designed with the intention of providing an increased sensitivity to the inducer and a higher maximum expression level of a gene product. When the cell culture was propagated at the nominal 1:100 dilutions no hysteresis was observed, which was reported as such in the past [4].

A closer analysis of the gene network shown in Figure 1 suggests that this circuit satisfies the requirements for bistability, i.e. cooperative positive feedback and high gene expression rates. It is qualitatively similar to networks that display hysteresis [9,13], and an analysis of the biochemical model provided a prediction on the experimental conditions (i.e. inducer levels) within which we could expect to see hysteresis. However, using the conventional 1:100 dilution procedure for propagating the induced culture we repeatedly failed to observe significant hysteresis.

We therefore hypothesized that the observation of hysteresis was obscured by some phenomenon specific to the culture propagation conditions. We hypothesized that a) there should be some batch propagation conditions under which hysteresis would be observable, b) the lack of hysteresis in other cases would be a result of a rapid dilution of the gene product that occurs during exponential growth after resuspension (causing some cells to switch OFF), and c) hysteresis could be further confounded due to the difference in growth rates between the ON and OFF phenotypes. Presumably, if the OFF phenotype grew at a faster rate owing to a smaller burden on the cell, the OFF phenotype would outcompete the ON phenotype. In other words, an engineered gene circuit would impose an additional metabolic burden on the host cell and would constitute a strong selective pressure against itself within a population.

The design of the experiment therefore involved determining the precise range of inducer concentrations that would result in hysteresis, independent determination of growth rates of ON and OFF phenotypes, modification of the dilution process as a way to control for the differences in growth rates between the ON and OFF phenotypes and determining the relationship between the dilution process and observed hysteresis.

## Methods

### Plasmids for the positive feedback gene network

The gene network is implemented on two separate plasmids. The ‘trigger’ plasmid pGN12 harbors an engineered variant of the *luxR* gene (denoted *luxR* Δ), which is autoinducer independent. The *luxR* Δ is placed under the control of an anhydrotetracycline (aTc)-inducible promoter *P*_*LtetO*-1_. The feedback plasmid pGN68 consists of a copy of green fluorescent protein (GFP) and another copy of *luxR* Δ both under the control of the *luxI* promoter *PluxI* in a bi-cistronic configuration. Details of the plasmid construction including PCR primers, detailed construction steps and validation have been previously published [4]. Relevant characteristics of the plasmids are recapitulated in Table 1 and recounted here.

**Table 1:** **Plasmids used in this study [**[4]**]**

**Plasmid**	**Relevant characteristics**
pGN12 (trigger)	*kan*^*r*^*P*_*LtetO*-1_ – *luxR* Δ*ori* p15A
pGN23 (intermediate construction)	*cm*^*r*^*P*_*luxI*_*ori* ColE1
pGN69 (intermediate construction)	*cm*^*r*^*P*_*luxI*_ – GFP[*tagless*] *ori* ColE1
pGN68 (feedback)	*cm*^*r*^*P*_*luxI*_ – GFP[*tagless*]
	– *luxR *Δ*ori *ColE1

The plasmid pGN12 is a derivative of the plasmid pPROTet.E 6xHN (Clontech, Mountain View, CA). The ColE1 origin of pPROTet.E 6xHN was replaced with the p15A origin from pZA34-luc [24] using the restriction sites XbaI and SacI, and by swapping the chloramphenicol resistance with kanamycin resistance from pZE21 [24]. The *luxR* Δ gene is a deletion mutant of the *luxR* gene from *Vibrio fischeri*. (referred to as *luxR*Δ_2-162_) by Nistala et al. [4]. It was obtained PCR amplification using the plasmid pLuxRI2 as a template. The native **luxR** protein is a 230 aa chain which is allosterically activated by the autoinducer acyl homoserine lactone (AHL). **luxR**Δ_2-162_ is a truncated form of that protein that retains only the DNA-binding, C-terminal domain of the protein. Deleting the N-terminal domain prevents it from interfering with DNA binding and yields an AHL-independent transcription activator [4,25,26]. The *luxR* Δ_2-162_ PCR product was digested with EcoRI and SalI and sub-cloned into the EcoRI and SalI cut-sites of the modified pPROTet.E plasmid described above, yielding pGN12.

The pGN68 plasmid was also based on pPROTet.E. The *luxI-GFP* transcriptional fusion was amplified using the promoter region of the plasmid p*lux*GFPuv [27] as a template. The resulting fragment was cloned into pPROTet.E using the restriction sites EcoRI and AatII yielding plasmid pGN23. The green fluorescent protein was amplified from pPROBE-*gfp*[tagless] [28]. The resulting fragment was then cloned into the EcoRI and BamHI restriction sites of the pGN23, yielding the plasmid pGN69. Subsequently the *luxR* Δ_2-162_ PCR product described above was digested with BamHI and NotI and sub-cloned into pGN69 yielding pGN68.

A schematic of the gene circuit is shown in Figure 1. The inducer aTc prevents the tetracycline-induced transcriptional repressor TetR from binding to the P_*LtetO*-1_ promoter through allosteric interaction thereby relieving repressed transcription from the trigger plasmid. The inducer aTc is a non-toxic analog of tetracycline. It does not inhibit cell growth at exposures of less than 100 ng/ml [4]. The transcription of *luxR* Δ proceeds in a manner dependent on the level of aTc induction, but independent of cell density. The gene product **LuxR*Δ*** promotes its own expression by binding to the *PluxI* promoter on the feedback plasmid and constituting a positive feedback. GFP expressed from the same promoter is used to measure the transcriptional activity of the *PluxI* promoter.

### Bacterial strains and growth conditions

All cultures were grown in Luria-Bertani (LB) broth (tryptone: 10 g/L, yeast extract: 5 g/L, and NaCl: 10 g/L). The cultures were incubated at 37 °C. The *E. coli* strain used was GN100 (F–*ilvG rfb-50 rph-1*Δ*envZ* :: FRT attB_λ_::[P_*N*25_ – *tetR lacI*^*q*^*spcR*]). Strain GN100 is a derivative of MG1655 with the chromosomal integration of the TetR/LacI expression cassette from DH5 αZ1. This strain constitutively expresses TetR thereby tightly repressing any genes under the control of the *P*_*LtetO*-1_ promoter in the absence of anhydrotetracycline (aTc). Details of the strain construction are published elsewhere [4]. The following antibiotics were added to the growth media: chloramphenicol (34 μg/mL) and kanamycin (40 μg/mL).

### Fluorescence assays

Before initiating experiments, cultures were grown overnight (∼12 h) from freezer stock in 2 mL of LB media with chloramphenicol and kanamycin. Subsequently, fresh media along with antibiotics was inoculated with cells from the overnight culture to a dilution of 1:100 to an optical density at 600 nm (OD_600_) of approximately 0.05. A Tecan infinite m200 96-well microplate reader (Männedorf, Switzerland) was used to measure optical density and fluorescent intensity (FI) of a 200 *μ*l sample, with excitation at 488 nm and emission at 520 nm and 9 spatially-distributed reads per well. The fluorescent measurements are reported using relative fluorescence units (RFU), defined as fluorescent intensity normalized by optical density. Experiments were performed using three samples in all cases. For kinetic measurements, fluorescent intensity and optical density measurements were taken every hour. Between measurements, the 96-well plate was agitated on the built in, 2 mm amplitude orbital shaker until approximately 3 m before the next measurement. The temperature of the well plate was maintained at 37±0.5 °C using the built-in temperature controller in the microplate reader.

All flow cytometry experiments were performed using a BD Biosciences LSR II flow cytometer (San Jose, CA). Flow cytometry samples were prepared by centrifuging and resuspending the entire culture in 1 ml ice cold PBS. The samples were stored on ice until measurements were taken. A minimum of 10,000 events were recorded using the high flow setting. Analysis of flow cytometry data was performed in FCS Express Version 3 and R®;, a statistical analysis environment.

### Experimental procedure

The hysteresis experiments were performed as follows: First, eight cultures were inoculated by diluting 1:100 from the overnight culture into 2 ml of fresh LB media and antibiotics. Cultures were grown for 2 h to an OD of ∼0.5 and then induced using aTc concentrations of 0, 0.1, 1, 5, 10, and 25 ng/mL. Three cultures were started using an aTc concentration of 25 ng/mL due to the large volume of this sample required for the subsequent hysteresis experiments.

Samples were thoroughly mixed and 200 *μ*L of the samples were placed in a 96-well plate for kinetic experiments. Cultures at induction levels of 0, 0.1, 1, 5 and 10 ng/mL aTc were replicated three times whereas the cultures induced at 25 ng/mL aTc had twenty seven 200 *μ*L samples placed into wells. 4 h after induction, one 200 *μ*L sample was taken from each induction level. We ensured the sample being taken had its OD and FI readings within ±1 standard deviation of the population average OD and FI. These samples were prepared for flow cytometry using the procedure described above. 12 h after induction one 200 *μ*L sample was again taken from each induction level and prepared for flow cytometry. These samples provided the baseline induction behavior for the circuit.

To test for hysteresis, the samples induced with 25 ng/ml aTc were diluted 12 h after induction. Four wells (800 *μ*L total) were pooled together, centrifuged at 15000 ×*g* and the supernatant was discarded. The sample was resuspended and washed in PBS, centrifuged and resuspended in an 800 *μ*L solution of LB, antibiotics, and 25 ng/mL aTc. This procedure was repeated on five sets of four wells, each set being resuspended in a solution of 10, 5, 1, 0.1, or 0 ng/mL aTc. Thus, six 800 *μ*L samples were created, all of which had a history of induction at 25 ng/mL aTc, followed by resuspending in a reduced concentration of the inducer corresponding to 0, 0.1, 1, 5, 10, and the control case of 25 ng/mL aTc.

From each 800 *μ*L sample, three 200 *μ*L samples were placed into microplate wells. These three samples formed dilution group A. The average number of cells per well from the original four wells that formed the 800 *μ*L sample was the same average number of cells per well for the new three wells. Thus, the dilution for group A was 1:1. (Note - in our case 1:1 dilution means 100% of the cells in the original suspension are retained in the solution. In theory, this implies that the solution is already at carrying capacity.)

From each 800 *μ*L sample, 60 *μ*L was mixed with a 540 *μ*L solution of LB, antibiotics, and the appropriate (i.e. consistent with the parent culture) concentration of aTc. This solution was mixed and placed into three microplate wells (200 *μ*L each). These three samples formed dilution group B. One-tenth of the average number of cells per well from the original four wells that formed the 800 *μ*L sample was the initial number of cells for these three new wells. Therefore the dilution for group B was 1:10, or 10% of the cells from the original sample were retained in the new solution.

Finally, from each 800 *μ*L sample, 6 *μ*L was mixed with a 594 *μ*L solution of LB, antibiotics, and the appropriate concentration of aTc. This solution was mixed and placed into three microplate wells (200 *μ*L each). These three samples formed dilution group C. The dilution for group C was 1:100, which means 1% of the cells from the sample were propagated further.

In summary, cells that were previously incubated at the high induction level (25 ng/mL aTc) were washed and resuspended at six different induction levels (0, 0.1, 1, 5, 10, and 25 ng/mL aTc). Each induction level contained samples inoculated using dilution ratios of 1:1, 1:10, or 1:100. There were three 200 *μ*L samples at each induction level and dilution for a total of 54 samples. These samples were grown in microplate wells during a kinetic experiment with measurements taken every hour.

Four hours after the dilution procedure, one 200 *μ*L sample was taken from each group at each induction level and prepared for flow cytometry. Twelve hours after the dilution procedure, another 200 *μ*L sample was taken from each group at each induction level and prepared for flow cytometry. All flow cytometry samples were analyzed at the same time, two hours after the collection of the last flow cytometry sample. In the interim, samples were stored on ice.

## Model

### Gene expression dynamics at the single cell level

To model the dynamics of the positive feedback system, we applied a model originally presented by Lewis and co-workers in 1977 [29-31]. The model demonstrates how positive feedback can lead to bistability and memory; it is presented in Equation 1.

(1) $$\frac{\text{dg}}{\text{dt}}=k_{1}s_{0}-k_{2}g+\frac{k_{3}^{2}g^{2}}{k_{4}^{2}+g^{2}}$$

In Equation 1, an inducer *s*_0_ activates transcription of a gene *g* in a linear fashion. The gene product *g* promotes its own expression, thereby establishing positive feedback within the system. The degradation rate of *g* depends on its own concentration and is assumed to be a linear function with degradation constant *k*_2_. The model constant *k*_1_ is the rate at which *g* is expressed by the inducer *s*_0_. The constant *k*_3_ governs the maximum expression level of the self-inducible promoter. Finally, *k*_4_ is the concentration of *g* needed to achieve half-maximum response of the self-inducible promoter. The Hill coefficient of 2 in the nonlinear term captures the cooperative behavior of the LuxR *Δ* dimer. The value of 2 for the Hill coefficient is derived from literature [32].

Equation 1 can be simplified to a dimensionless form as shown below: (See Additional file 1: section S1 for the derivation.)

(2) $$\frac{\text{dx}}{\mathrm{d\tau}}=s-\text{rx}+\frac{x^{2}}{1+x^{2}}$$

where, $s=\frac{k_{1}s_{0}}{k_{3}^{2}}$, $r=\frac{k_{2}k_{4}}{k_{3}^{2}}$, $x=\frac{g}{k_{4}}$, and $\mathrm{d\tau}=\frac{k_{3}^{2}}{k_{4}}\text{dt}$.

**Figure 2:**
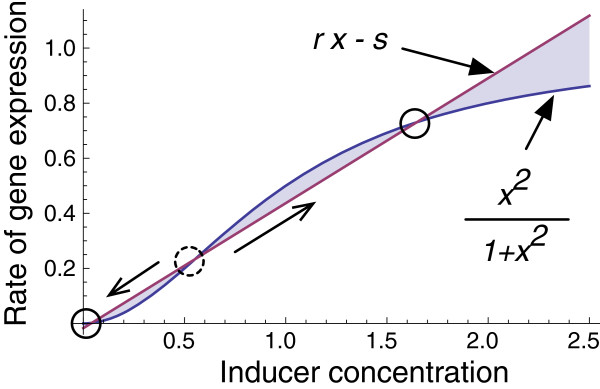
**Nullclines of Equation **2**.** The differential equation has as many as three points where the derivatives are zero. The two extreme points (shown by solid open circles) correspond to the bistable states. The intermediate point (dotted open circle) is an unstable point.

**Figure 3:**
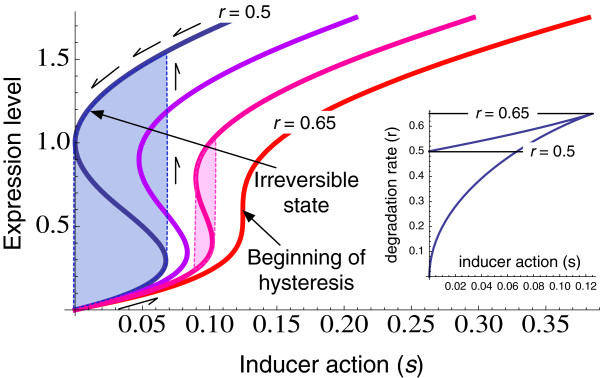
**Hysteresis in the dynamical system.** Figure shows the solutions for the gene expression level (*x*) as a function of *r* and *s*. Inset shows the region of bistability. For values of *r* between 0.65 and 0.5, the hysteretic behavior is reversible. For *r*<0.5, the system can get irreversibly locked into the ON state.

A graphical view of the dimensionless form of the differential equation is shown in Figure 2. Parametric analysis of the system represented by Equation 2, suggests that the system is capable of producing a bistable response for certain combinations of the dimensionless constants *r* and *s* that represent degradation and induction rates respectively. Moreover, as seen in Figure 3, a portion of the bistable space corresponding to values of *r*<0.5 produces irreversible bistability; once the expression rate is switched ON, it cannot be switched OFF by removing the inducer.

It is worth noting that the above biochemical model is a very minimalistic description of the synthetic gene circuit. It assumes a linear expression rate with respect to the inducer, which is a reasonable expression for the range of 0.02-10 ng/ml induction for our circuit. It represents transcription, translation and mRNA degradation as a lumped parameter *k*_3_. The model also assumes a first-order degradation rate that is simply proportional to the amount of the product at time *t*. Most of these model parameters are doubtless functions of the state of the cell and of the culture as a whole. We also explored a more complex model (see Additional file 1: section S1.3 to separate gene product degradation rates into individual dilution and decay parameters. However rather than pursue the route of building a complex, systems biology based approach to resolving the inconsistencies of experimental observation with the simplistic model, we approached the problem from the perspective of stochastic population dynamics in cell culture.

**Figure 4:**
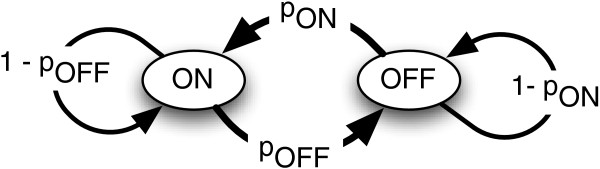
**The state transition diagram for a single cell.** The arrows between the ON and OFF states represent the probabilities of transition between the states per unit time.

We instead assume that the transition between the ON and OFF states of any given *E. coli* host cell can be modeled as a stochastic process. Assuming that the probability of a cell switching from OFF to ON per unit time is given by *p*_*ON*_ and the probability the cell switches OFF is given by *p*_*OFF*_, then the transitions can be represented by the state transition diagram as shown in Figure 4. Additionally, we assume that at steady state the transition probabilities *p*_*ON*_ and *p*_*OFF*_ are invariant with time, although we acknowledge that the transition probabilities will have their own dynamics that could have roots in the biochemical model of Equation 1.

### Population dynamics

We use a continuous-time logistic growth model (also known as the Verhulst-Pearl equation) as the foundation under nutrient-limiting conditions. The basic form of the model is as follows:

(3) $$\frac{\text{dN}}{\text{dt}}=\rho N\left(1-\frac{N}{K}\right)$$

Here, *N* is the population size, *ρ* is the growth rate and *K* is the carrying capacity, which denotes the maximum value *N* can attain. We treat the ON and OFF subpopulations as two distinct ‘species’ in neutral competition with each other, which may have their own growth rates and carrying capacities. Neutral competition implies that the populations merely compete for resources, but do not otherwise hinder or benefit each other. The total population *N* = *N*_*ON*_ + *N*_*OFF*_ is limited by the carrying capacity *K*. To capture the neutral competition between the ON and OFF phenotypes, we set up a system of autonomous differential equations as follows [33]:

(4) $$\begin{array}{lll} \frac{d}{\text{dt}}N_{\text{ON}} & =\rho_{\text{ON}}N_{\text{ON}}\left(1-\frac{N_{\text{ON}}+\alpha N_{\text{OFF}}}{K}\right)\; & \;\\ \frac{d}{\text{dt}}N_{\text{OFF}} & =\rho_{\text{OFF}}N_{\text{OFF}}\left(1-\underset{\text{Competition}}{\underset{︸}{\frac{N_{\text{ON}}+\beta N_{\text{OFF}}}{K}}}\right)\; \end{array}$$

Here, *N*_*ON*_ and *N*_*OFF*_ are population sizes of the ON and OFF phenotypes, *ρ*_*ON*_ and *ρ*_*OFF*_ are the respective growth rates and *K* is the carrying capacity. The coefficients *α* and *β* determine the nature of interaction between the ON and OFF subpopulations. For neutral competition, α = β = 1. For a discussion about competitive Lotka-Volterra models, see Additional file 1: Section S2.1 and [33].

The basic Lotka-Volterra competitive model does not cover the case where an organism changes from one ‘species’ to another. As shown in Figure 4, in our system any given cell may switch between the ON and OFF phenotypes with a given probability. Thus in the population dynamics model, the rate of change the ON or OFF population is dependent upon not only the intrinsic growth rate of the phenotype, but also on the change in the population through phenotype switching. If *p*_*ON*_ and *p*_*OFF*_ are the probabilities of turning ON and OFF respectively, then the total number of ON cells is the weighted sum of ON cells that remain ON and OFF cells that turn ON. Therefore we replace *N*_*ON*_ with (1-*p*_*OFF*_)*N*_*ON*_+*p*_*ON*_*N*_*OFF*_, and similarly *N*_*OFF*_ with (1-*p*_*ON*_)*N*_*OFF*_+*p*_*OFF*_*N*_*ON*_. Note that the probability terms *p*_*ON*_ and *p*_*OFF*_ cancel out in the competition term in the parenthesis for α=β=1.

Substituting the values for *N*_*ON*_ and *N*_*OFF*_, we therefore get the following system of coupled differential equations:

(5) $$\begin{array}{lll} \frac{d}{\text{dt}}N_{\text{ON}} & =\rho_{\text{ON}}\left((1-p_{\text{OFF}})N_{\text{ON}}+p_{\text{ON}}N_{\text{OFF}}\right)\; & \;\\ & \;\times \left(1-\frac{N_{\text{ON}}+N_{\text{OFF}}}{K}\right)\; & \;\\ \frac{d}{\text{dt}}N_{\text{OFF}} & =\rho_{\text{OFF}}\underset{\mathrm{Phenotype}\;\text{switching}}{\underset{︸}{\left(p_{\text{OFF}}N_{\text{ON}}+(1-p_{\text{ON}})N_{\text{OFF}}\right)}}\; & \;\\ & \;\times \underset{\mathrm{Neutral}\;\text{competition}}{\underset{︸}{\left(1-\frac{N_{\text{ON}}+N_{\text{OFF}}}{K}\right)}}\; \end{array}$$

The above equations are autonomous - i.e. the growth rates do not depend upon time. An analysis of the model is shown in Additional file 1: Section S2.

## Results and discussion

### Determining rate constants and predicting hysteresis

**Figure 5:**
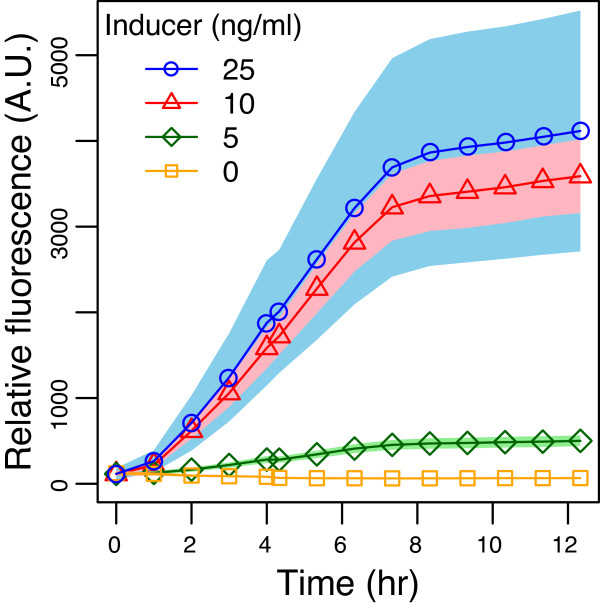
**Expression of GFP with time for various inducer levels.** Open circles, triangles, diamonds and squares correspond to 25, 10, 5 and 0 ng/ml induction. Expression levels corresponding to 1 and 0.1 ng/ml were not significantly different from the 0 ng/ml expression level, and are omitted from the plot. Shaded regions outline two standard deviations around the mean. Additional file 1: Figure S6 shows that the growth rates for the different inducer levels were statistically identical.

*E. coli* GN100 cells cotransformed with the positive feedback circuit (pGN12 and pGN68) were induced with 0, 0.1, 1, 5, 10 and 25 ng/ml aTc. The cells were grown on a 96 well plate, their optical density and fluorescence levels were recorded. The results are shown in Figure 5.

For the data in Figure 5, numerical derivatives were computed and fitted to Equation 1 to compute the rate constants *k*_1_ through *k*_4_. The nonlinear regression analysis and model constants are detailed in Additional file 1: Section S3. For the various induction levels, the corresponding dimensionless *r* and *s* parameters were calculated. Significantly, the data suggests that the fitted value of *r* is 0.26 ±0.06, which is well below 0.5, the level below which the system should display irreversibly hysteretic behavior. The value of *r* predicts that the switching threshold for the system is at approximately 2.4 ng/ml, which agrees with the data; no GFP expression is seen for 1 ng/ml, but the expression becomes apparent at 5 ng/ml. The biochemical model predicts that once the circuit is switched ON (induced at > 2.4 ng/ml), the GFP expression should retain the memory of the induction event even after removal of the inducer. We then proceeded to look for the conditions under which hysteresis should be apparent.

### Searching for hysteretic behavior

Cells containing the positive feedback-based gene amplifier were grown at a high induction level (25 ng/mL aTc) for 12 h to a steady-state expression level. These cells were then centrifuged, washed, and resuspended into media at various inducer concentrations (0, 0.1, 1, 5, 10, and 25 ng/mL) and three dilutions (1:1, 1:10, and 1:100).

Dilutions were performed 12 h after inoculation to provide sufficient time for gene product concentration to reach steady-state. The slight linearly increasing trend shown in Figure 5 is due to the cultureÕs slight decrease in optical density after saturation, and not due to any significant change in GFP concentration within living cells. Dilutions were performed in the stationary phase because this is when GFP concentration is at steady-state.

**Figure 6:**
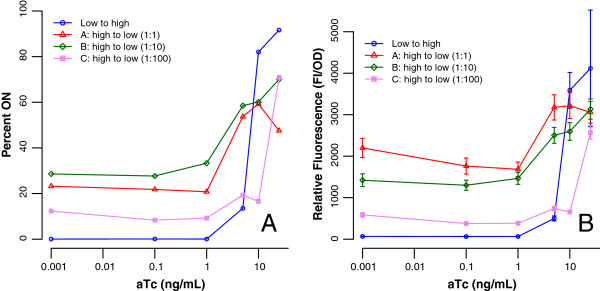
**Hysteresis is affected by the dilution used for subculture.A**: Hysteretic behavior in cell populations resuspended using three dilutions. Open circles show the increase in GFP intensity as a function of inducer concentration. Open triangles, diamonds and squares indicate three different dilutions. All three dilution groups showed expression levels significantly greater than the cells with no induction history at low inducer concentrations. Cell populations resuspended using the lowest dilution (Curve A with 1:1 dilution) generally showed the highest expression levels upon reaching steady-state, and populations resuspended using the highest dilution (Curve C, 1:100) showed the lowest. Therefore, the overall magnitude of the population-level hysteresis depends on the dilution used during resuspension, and hysteresis may not be observable within a population if very high dilutions are used during resuspension. *Note: error bars represent ±2 standard deviations.***B**: Hysteresis quantified in terms of proportion of cells in the ON state. The trends for the three dilutions closely resemble trends seen in relative fluorescence.

Figure 6A shows the steady-state hysteretic expression behavior measured as relative fluorescence (RF), of cell populations resuspended using the three different dilutions and grown for 12 h. Cell populations with no induction history (low to high) exhibit classic ‘switch-like’ behavior, consistent with previously reported values [4]. Cell populations with a high induction history, and subsequent reduction / removal of inducer (Curves A, B, and C) display varying levels of hysteresis: the expression level at lower inducer levels is significantly higher than cells having no prior history of induction. A sustained, relatively high level of expression is also seen in the cases when cells are resuspended in media with no inducer, indicating that the effect of initial induction is irreversible as predicted by the model for gene expression.

However, it is important to note that the amount of hysteresis seen in the experiments seemed to be related to the dilution level. Cell populations that were not diluted (i.e. a 1:1 resuspension) showed the highest level of retained memory, whereas the 1:100 subculture shows almost no retained memory; a higher level of dilution resulted in a lower level of apparent hysteresis. This observation cannot be predicted from the simple biochemical model and has not been previously reported to the best of our knowledge.

To determine whether the reduction in relative fluorescence was due to the reduction in the average fluorescence per cell, or the reduction in the proportion of cells displaying the ON phenotype, the cell populations of Figure 6A were also analyzed by flow cytometry. Figure 6B shows the percentage of cells that are in the ON state after resuspending at the lower inducer levels. The primary change among the three dilution groups is the proportion of cells in each population that are ON, although we observed that there is a small reduction in the amount of expression per cell (i.e. mode of the distribution) for the higher dilutions. (See Figure S8 in Additional file 1 as an example). This reduction may be due to dilution of GFP; the 1:1 culture has limited capacity to dissipate the gene product by way of dilution whereas the magnitude of the dilution rate in 1:10 and 1:100 cultures is likely greater.

When hysteresis is quantified as the proportion of cells within a population that are ON, as in Figure 6B, the trends for each dilution group are similar to when hysteresis is quantified using relative fluorescence, as in Figure 6A. However, there is a small discrepancy between the relative magnitudes of hysteresis between each dilution group. Figure 6A shows that group B (1:10) shows a lower value for relative fluorescence than group A (1:1), but Figure 6B shows that group B (1:10) has a higher proportion of ON cells than group A (1:1). This is because although dilution group A had a lower proportion of ON cells within each population compared to group B, the median fluorescence level for the ON cells was higher. See Additional file 1: Section S4 for further clarification.

### Determining bounds on the switching probabilities

Figures 6A and 6B show that the appearance of hysteresis is related to the dilution factor, and not just on the biochemical model of gene expression. It appears that the dilution factor masks the hysteresis to a significant extent, such that at subculture dilutions of 1:100, hysteretic behavior is almost completely obliterated.

If the ON or OFF state of the gene circuit were stably heritable, we should expect the same proportion of ON cells in each of the three dilution factors. However, if either 1) the OFF phenotype grows faster than the ON phenotype, or 2) the gene circuits switch to the OFF phenotype by dropping below the unstable point shown in Figure 2, then the population would trend towards an increasing OFF phenotype. To determine whether OFF phenotypes grew faster than the ON phenotypes, we sorted a population of cells induced at 5 ng/ml. We used the lowest practical concentration of aTc to produce a bimodal distribution so as to have roughly equal numbers of individuals in the ON and OFF phenotypes. Additionally, the low inducer concentration also minimizes imposing any additional burden (a documented cause of loss-of-function mutants in engineered circuits [34]) on the cell due to excessive GFP production.

**Figure 7:**
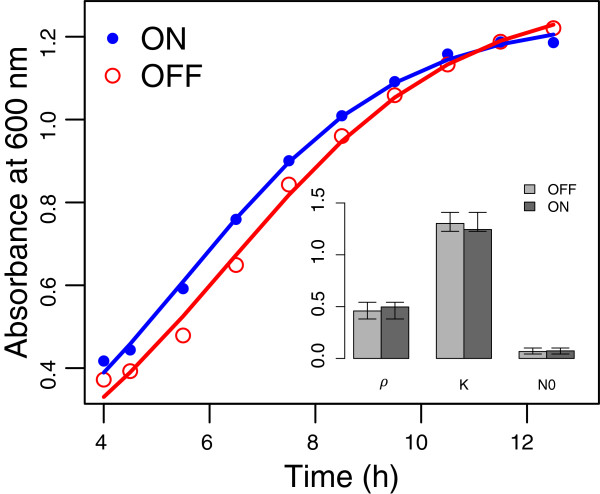
**Growth data for sorted ON and OFF subpopulations.** Open circles show the growth of the OFF population, while solid circles denote the ON subpopulation. The curves denote the model fits. Inset: Statistical analysis on the fitted parameters shows that the parameters are not statistically significantly different.

The sorted ON and OFF phenotypes were independently grown for 12 h in fresh media containing 5 ng/ml of aTc, in order to maintain identical inducer levels, and the culture absorbance was recorded. The logistic growth model was fit to the growth data. The parameters for the growth model and the fitted curves are shown in Figure 7.

Figure 7 shows that the logistic model from Equation 3 is a reasonable approximation of the growth data for the individual subpopulations, and that the *ρ*, *K* and *N*_0_ parameters for the ON and OFF subpopulations are not significantly different. The growth rate *ρ* is in fact somewhat smaller for the OFF population, although the carrying capacity *K* is not statistically significantly larger.

In addition to growth rates, flow cytometry was used to characterize the phenotypic distribution at 4 h after sorting (corresponding to the fastest growth rate) and after 12 h, at the end of the experiment. The results of the distribution were as follows: In case of the OFF population at the end of 12 h only 3% of the cells had turned ON. We interpret data as the fraction of the population which cannot be turned ON at an induction level of 5 ng/ml.

For the initially ON population, after 4 h, only 1% of the ON cells showed any expression. At the end of 12 h, 72% of the cells showed fluorescence expression. This number is significantly higher than the ∼15% of cells that are ON after an induction of the naïve cells at similar (5ng aTc) induction levels. The overall higher number suggests that the observation of 1% ON cells after 4 h is a temporary effect. There are two possible reasons why the gene expression appears to temporarily vanish. Firstly, an induction level of 5 ng/ml is only slightly higher than 2.4 ng/ml, the predicted location of the OFF to ON transition threshold. Thus it is likely that at that concentration the positive feedback delays the onset of expression and most cells are in the process of turning ON at 4 h. The second possibility is a likely greater magnitude of growth-mediated dilution in the early stages of the culture that retards the accumulation of the **LuxR**Δ and GFP gene products. Although the possibility of very different protein degradation / expression rates in different stages of a batch culture well-known in *E. coli*[35-37], its importance to the characterization and dynamics of synthetic gene circuits is largely overlooked.

While the cell sorting experiment above was not designed to be a test for hysteresis, but rather to measure growth rates for the different ON and OFF populations, the results suggest that the increased dilution of the transcription factors during the exponential growth phase can significantly change the observed levels of gene expression and present a source of inconsistency with the simplistic biochemical model.

Analysis of the stochastic behavior of the cells induced at 5 ng/ml provides a lower bound on the ON to OFF transition probability of the gene circuit. As a comparison, when cells were induced at 10 and 25 ng/ml aTc concentrations and resuspended in a 1:1 dilution we observed that between 47 and 59% of the cells retained the ON phenotype, as compared to 72% when induced at 5 ng/ml. It is conceivable that at the higher induction levels of 10 and 25 ng/ml the likelihood of a cell switching to OFF may be higher owing to the additional metabolic burden imposed by the higher induction rate. However it is difficult to directly test this hypothesis because at higher induction levels the majority of the cells are ON to begin with and it is not possible to get a large enough sample of the OFF phenotypes to investigate comparative growth rates.

**Figure 8:**
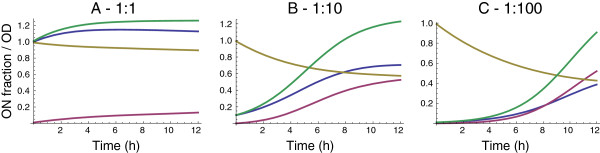
**Numerical solution showing the ratio of ON cells.** The magenta and blue lines represent the OFF and ON phenotypes respectively. The solution assumes that the culture is started with a pure ON population, and cells switch to OFF over time. The numerical values for the growth rates and carrying capacities of the ON and OFF phenotypes were obtained from the model shown in Figure 7. The green line shows the size of the total population, while the beige curve starting at 1 shows the proportion of ON cells in the total culture. Panels **A-C** correspond to the three different dilution ratios tested. The final value of the ON fraction decreases as the dilution increases. In the 1:100 dilution case, the ON fraction is approximately 42%, while that in the 1:1 case is about 85%.

Assuming the lower bounds on switching probability rates of $p_{\text{ON}}^{\prime }=0.03$, and $p_{\text{OFF}}^{\prime }=0.28$ (from the flow cytometry data) we then proceeded to model a mixture of ON and OFF phenotypes using the modified Lotka-Volterra competitive model as shown in Equation 5. By using reasonable approximations on the initial distributions of ON and OFF populations and the starting concentration of the inoculum it is possible to solve the system of differential equations in 5 to model the effect of dilution on the culture. The model captures the dilution effect as seen in Figure 8.

### Interpretation of the competitive model and model estimates

Although we used the values of $p_{\text{ON}}^{\prime }=0.03$ and $p_{\text{OFF}}^{\prime }=0.28$ in modeling the competition based on Equation 5, it is important to note that there is a difference between the estimates denoted with the primes and the terms *p*_*ON*_ and *p*_*OFF*_ in Equation 5. Specifically, the estimates denote the total proportion of cells that have switched phenotypes at the end of an observational interval of 12 h, whereas the model considers *p*_*ON*_ and *p*_*OFF*_ as instantaneous, differential probabilities, which are obtained in the limit as the observation interval goes to zero.

In addition to the stochastic interpretation, the switching probabilities are analogous to hazard rates seen in reliability theory because the probability of an OFF to ON transition is very close to zero. In other words, the OFF state is an absorbing state representing the eventual failure of the gene circuit. In our model, the hazard rate may be interpreted as the instantaneous conditional probability that a cell switches its phenotype from state A to another state B in the instant after time *t* given that it persisted in state A until time *t*. It has units of *s*^-1^. Our model implies the simplest case of a constant hazard rate, which implies an exponentially decreasing survival rate of the ON phenotypes which is readily seen in Figure 8.

An important, but unsurprising consequence of both the stochastic or survival models for the population is worth reiterating: Over a long enough observation interval the expression of GFP at the population level will degrade to zero if there is no selection pressure acting in favor of the circuit. It is known that the greater the metabolic burden on the circuit, the shorter will be its functional life [34]. The eventual degradation of the circuit implies the following question: Does hysteresis even exist in our system? In other words, can we distinguish between the noisy, stochastic switching between the ON and OFF phenotypes in a bistable circuit from the gradual loss-of-function failure due to evolutionary instability? We think the answer is yes, based on the results of the cell sorting experiment where cells were induced at 5 ng/ml. Cells that were ON were regrown in fresh media. After 12 h 72% of the cells were ON. This number agrees very well with the expected half life of tagless GFP of 24 hours [3], which would imply an ON rate of about 70% after 12 hours. Also, this number is significantly higher than the ∼15% of cells that are ON after an induction of naïve cells at similar conditions. Both experiments have the same noisy circuit, so in the former case, significantly higher proportion of ON cells may be attributed to deterministic hysteresis.

We note that neither the simple biochemical model nor the population dynamics overlayer fully capture the complex network dynamics. The observation that only 1% of the cells subcultured from the ON phenotype were seen to express GFP 4 h after sorting suggests that cell growth seems to occur with a higher priority than heterologous protein expression in the exponential phase of the culture, thus questioning the assumption of a linear dilution / decay rate in the biochemical model (Equation 2). Indeed, the decay rate *k*_2_ used in the equation is a composite rate consisting of dilution due to cell division (which halves the concentration of LuxR in the cell) and the intrinsic decay rate of the dimer itself. The switch to a fast growth rate could dramatically change the composition of *k*_2_. This coefficient *k*_2_ is the gateway to fuller integration between the population dynamics and gene expression models. We explored the possibility of reworking the biochemical model to decouple *k*_2_ into two independent dilution and decay terms (See Additional file 1 section S1.3. The resulting model is difficult to analyze, and better experiments will have to be devised to quantify and support the notion of changing decay rates. We leave this avenue open for future investigation.

The source of discontinuity in dilution / decay rates may be found in the different sigma factor regimes during the growth and stationary phases of a culture. As far as we can tell, no systems biology models currently account for stationary phase behavior, although there are validated theories for stationary cultures under continuous culture conditions [22,23]. The lack of a systems biology model for stationary response represents a significant gap in the current state of knowledge. To build a more accurate model we would not only have to modify the decay to be a function of the state of the culture, but would also have to account for the time dependence of the transition probabilities. In the interim however, a probabilistic measure as described here can recapitulate the effect of dilution rates in the population dynamics model.

A competitive population dynamics model supports the idea that in a limited resource environment the so called *K-strategist* (competitor that shows an increased resource efficiency) will always outcompete other competitors, even if the competitors display a faster growth rate and even if the efficiency (the *K* value or carrying capacity) of the K-strategist is only marginally higher. In our system, the primary determinant of the increasing OFF population was the switching rate between the ON and OFF phenotypes; any differences in the growth parameters between the phenotypes were smaller than the resolution of our experimental techniques. If the switch from ON to OFF may be interpreted as a K-strategy, since the OFF phenotypes no longer expend resources to express GFP, then the loss of function is consistent with accepted ideas in population dynamics.

### Conclusions

Our experiments showed that the positive feedback-based gene amplifier was capable of producing a hysteretic response to a stimulus in a single cell, but the observation of hysteresis was significantly modulated by the stochastic transition to the OFF phenotype, and this effect was even higher when the cells were subjected to changes in growth conditions through subculturing. The dilution ratio used to propagate the cell culture determined the failure rate of the gene expression circuitry at the population level beyond the failure rate of the circuit at the single cell level. The higher the dilution rate, the faster was the observed failure.

More generally, in applications where a certain behavior is expected from a cell population (such as whole cell biosensors or cell-based computers), nonlinear changes in degradation and dilution of the gene products can lead to failure with respect to the desired operation of the gene circuit. The above experiments are an illustrative example of the interplay between gene expression and population dynamics. This interplay is expected to be complex and cannot be ignored if we are to design and build non-trivial, robust genetic circuits.

Stochasticity in gene expression and adaptation all but ensure that synthetic gene circuits will have a finite operational lifespan unless their operation is constantly under a positive selection pressure. Absent directed evolution, tools that allow the bioengineer to predict the durability and reliability of gene circuits will be a critical to advance synthetic biology towards industrial biotechnology. Population dynamics models provide an entry point into quantifying the probabilistic behavior of a population of cells over time scales of the order of the replication rate of the organism, which is intermediate between gene regulatory processes and the slower evolutionary processes. As such they serve an important need in modeling the performance over time scales relevant to applications of synthetic biology such as bioprocessing and metabolic engineering.

## Competing interests

The authors declare that they have no competing interests.

## Authors’ contributions

PP and KB designed the experiments. PP conducted the laboratory experiments. PP and KB developed the models and conducted the statistical analyses and contributed to the writing of the manuscript. Both authors read and approved the final manuscript.

## About the authors

Phillip Poisson received his bachelor’s degree in mechanical engineering from the University of Michigan at Ann-Arbor, and his master’s degree in mechanical engineering from the University of Illinois at Urbana-Champaign. He currently works in Research and Engineering at Kimberly-Clark Corporation in Neenah, Wisconsin.

Kaustubh D. Bhalerao received his PhD in Biological Engineering in 2004 from the Department of Food, Agricultural and Biological Engineering at The Ohio State University in Columbus, Ohio. He joined the faculty at the Department of Agricultural and Biological Engineering at University of Illinois as an assistant professor in 2005. He is currently an associate professor. His research interests include understanding evolution from the context of biological engineering, and the impact of nanotechnology on the environment. He is a member of two editorial boards.

## Supplementary Material

Additional file 1 Supplementary materials detailing the derivation, dynamical analysis and statistical fits of the dynamical systems models described in the main text. Click here for file
