# Supplementary material for: Hidden hysteresis – population dynamics can obscure gene network dynamics
Source: J Biol Eng. 2013 Jun 24;7:16. doi: 10.1186/1754-1611-7-16 (PMC3700772; doi:10.1186/1754-1611-7-16)
Supplement: Additional file 1 — Supplementary materials detailing the derivation, dynamical analysis and statistical fits of the dynamical systems models described in the main text. [file 1754-1611-7-16-S1.pdf]

Supplementary Materials: Hidden hysteresis population dynamics can obscure gene network dynamics

Phillip Poisson   Kaustubh D. Bhalerao*

S1 Dynamical systems analysis for the behavior of gene expression at the single cell level

S1.1 Simplification of the full model to the dimensionless form

The Lewis model for gene expression and its reduced, dimensionless form are reproduced from the main text here:

$$\frac{dg}{dt} = k_1 s_0 - k_2 g + \frac{k_3^2 g^2}{k_4^2 + g^2} \tag{S1}$$

Dividing the non-linear term involving g on the RHS by k_4^2:

$$\frac{dg}{dt} = k_1 s_0 - k_2 g + \frac{(k_3^2/k_4^2) g^2}{1 + (g^2/k_4^2)} \tag{S2}$$

Setting $x = g/k_4$, we get:

$$k_4 \frac{dx}{dt} = k_1 s_0 - k_2 k_4 x + \frac{k_3^2 x^2}{1 + x^2} \tag{S3}$$

*corresponding author. 1304 W Pennsylvania Ave, Urbana, IL 61801, bhalerao@illinois.edu

Dividing throughout by k_3^2 we get:

$$\frac{k_4}{k_3^2}\frac{dx}{dt} = \frac{k_1 s_0}{k_3^2} - \frac{k_2 k_4 x}{k_3^2} + \frac{x^2}{1+x^2} \tag{S4}$$

Substituting $d\tau = \frac{k_3^2}{k_4}dt$, $s = \frac{k_1 s_0}{k_3^2}$, and $r = \frac{k_2 k_4}{k_3^2}$, we reduce equation S1 to the non-dimensionless form as shown below:

$$\frac{dx}{d\tau} = s - rx + \frac{x^2}{1+x^2} \tag{S5}$$

S1.2 Analyzing the dynamical system

The stable points of Equation S5 occur when $\frac{dx}{d\tau} = 0$, or when $\frac{x^2}{1+x^2} = -(s - rx)$. Figure S1 can be used to visualize the stable points of Equation S5.

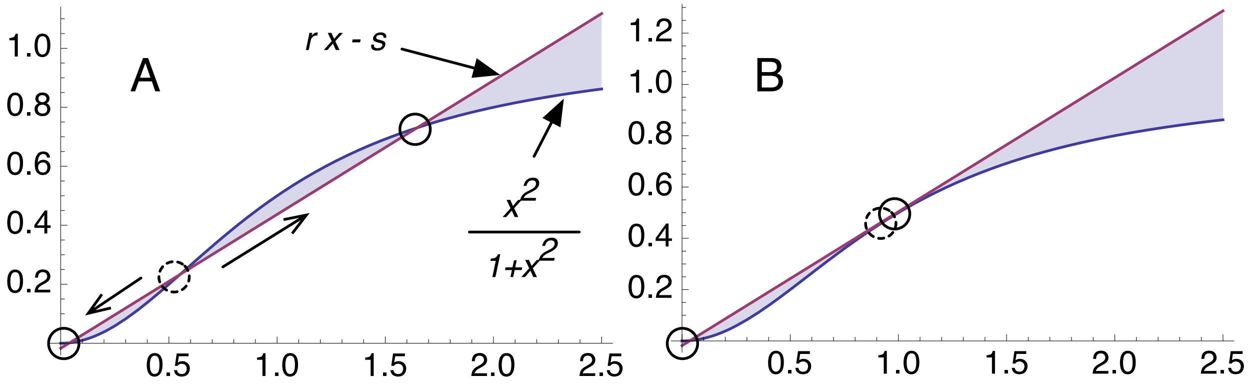

Figure S1: (A) Equation S2 can have as many as three roots. The intermediate solution (dotted circle) is unstable. (B) Limiting case where two of the three solutions are identical.

Depending on the specific values of r and s, we can get between zero and 3 solutions. When three solutions exist, as shown in Figure S1(A), the two outside solutions are stable, while the middle solution is unstable, making the system bistable.

The boundary condition for bistability arises when the unstable solution moves towards one of the stable points. When the two solutions coalesce, the slope of the two curves, as well as the ordinates are equal. Mathematically:

$$rx - s = \frac{x^2}{1+x^2} \text{ and} \tag{S6}$$
$$\frac{d}{dx}\frac{x^2}{1+x^2} = r \tag{S7}$$

Solving these above equations we obtain the following expressions for r and s parameterized in x:

$$r = \frac{2x^2}{(1+x^2)^2} \tag{S8}$$
$$s = \frac{x^2(x^2-1)}{(1+x^2)^2} \tag{S9}$$

The above equations can be plotted as a parametric plot to visualize the dependence of the system behavior on r and s as seen in Figure S2.

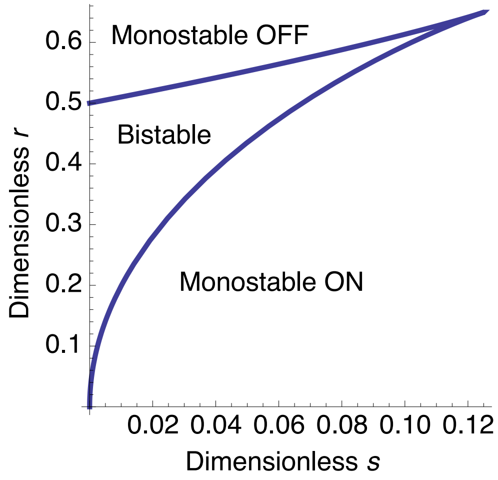

Figure S2: The parametric r–s space showing the region in which the circuit operates in a bistable manner.

Setting Equation S5 to zero, substituting different values of r and solving for x we get the behavior of the solutions of Equation S5. These solutions are plotted in Figure S3 below: The key conclusion from Figure S3 is that the system is capable of hysteretic behavior.

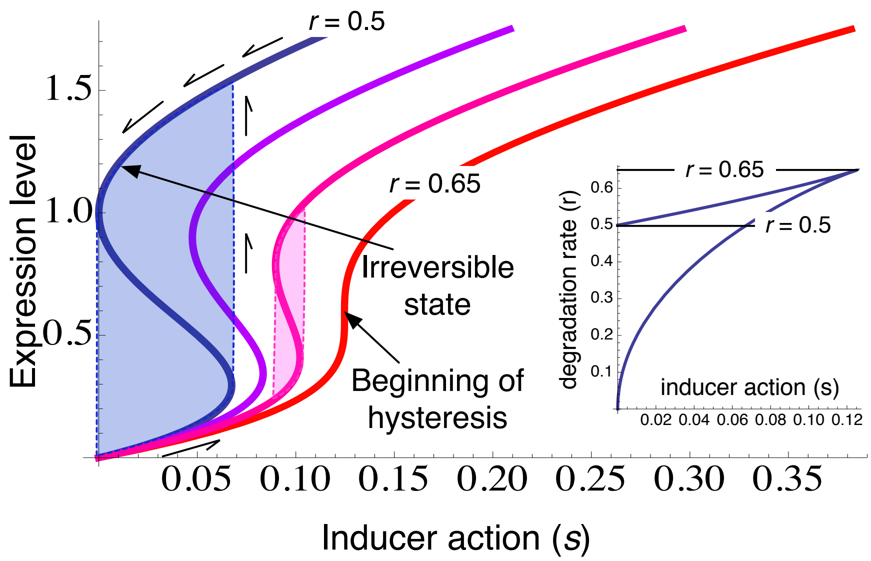

Figure S3: The roots of Equation S5 as a function of r and s. For $r < 0.65$, system displays hysteresis. For $r < 0.5$, the hysteresis is irreversible. Inset shows the corresponding values on the r–s parametric space.

For values of $0.5 < r < 0.65$, there exist two stable solutions of x for a range of values of s, as shown by the shaded regions in Figure S3. Once the inducer action s crosses a threshold, the corresponding value of x jumps to a higher value. Hereafter, reducing the value of s below the threshold does not allow the value of x to return to the previous lower solution. Further reduction in s may prompt the system to fall back to the lower state. However, for values of $r < 0.5$, system irreversibly locks into the higher state. In other words, the state of the system depends upon the past history of induction. If the inducer value exceeded the OFF to ON switching threshold of s, the system will be locked in the ON state.

S1.3 Is a more complex model warranted?

One reasonable criticism of our approach is that the biochemical model we have chosen is far too simple, and that a complex model may be warranted to better capture the behavior of the expressed genes. There are two ways in which our model can be

improved. Firstly, we can assume a bipartite degradation term that separately accounts for decay and dilution. Moreover, since we re modeling our culture as a batch process we can assume that the dilution rate decreases with time as the cell reproduction rate decreases. The decay rate can be assumed linear with respect to the gene product, while the dilution rate can be approximated as an exponential decay.

Secondly, we can reinterpret the term x equation S5 to be the concentration of LuxR, rather than GFP. The expression of GFP can have its own induction rate proportional to x and degradation and dilution rates proportional to its own concentration. Thus the equation in S5 instead becomes a system of coupled differential equations as follows:

$$\frac{dx}{d\tau} = s - \underbrace{r_1 x}_{decay} - \underbrace{e^{r_2 x}}_{dilution} + \frac{x^2}{1+x^2} \quad \text{(S10)}$$

$$\frac{dG}{d\tau} = s_g x - r_1 g - e^{r_2 g} \quad \text{(S11)}$$

However this system is difficult to solve numerically; we weren't able to explore its behavior due to the absence of solutions of this very stiff system. Dropping the exponential dilution terms we get a system that can be solved, but one which can be approximated satisfactorily with the much simpler form in S5. We therefore do not pursue this line of reasoning and instead move to a population-level model where the gene expression is abstracted into a ratio of ON to OFF cells.

S2 Modeling a mixture of cells

S2.1 A modified Lotka-Volterra Competitive Growth Model

When two populations of cells having their own growth rate (ρ), carrying capacity (K) and initial starting concentration (N) values are mixed together, the general form of the

logistic growth model needs to be modified slightly. The modulation of the growth rate $\frac{dN_I}{dt}$ for each of the i organisms is due to the total population of cells $\sum_i N_i$ in the culture. For $i \in \{ON, OFF\}$ the growth of each individual population can be modeled according to the system of equations (Equations 4 in the main text), as follows:

$$\frac{d}{dt}N_{ON} = \rho_{ON}N_{ON}\left(1 - \frac{N_{ON} + \alpha N_{OFF}}{K}\right)$$
$$\frac{d}{dt}N_{OFF} = \rho_{OFF}N_{OFF}\left(1 - \underbrace{\frac{N_{ON} + \beta N_{OFF}}{K}}_{\text{Competition}}\right) \tag{S12}$$

The coefficients α and β determine respectively how much the presence of the OFF population impacts the growth rate of the ON population and vice versa. The values for the coefficients can be positive or negative, which can be used to model trophic dependencies in an ecosystem.

The following table shows the various values of α and β and the ecological interactions they represent. In our case, the ON and OFF phenotypes are in neutral competition for growth media resources.

Table S1: Ecological interactions in a competitive Lotka-Volterra model

α	β	Interaction
1	1	Neutral competition for resources only
$\alpha < 1$	$\beta < 1$	Mutualism / synergy between species
$\alpha > 1$	$\beta > 1$	Mutual inhibition
$\alpha > 1$	$\beta < 1$	Parasitism by the OFF phenotype at the cost of the ON phenotype
$\alpha < 1$	$\beta > 1$	Parasitism by the ON phenotype at the cost of the OFF phenotype

$$\frac{d}{dt}N_{ON} = \rho_{ON}\left((1-p_{OFF})N_{ON} + p_{ON}N_{OFF}\right)\left(1 - \frac{N_{ON}+N_{OFF}}{K}\right)$$
$$\frac{d}{dt}N_{OFF} = \rho_{OFF}\left(p_{OFF}N_{ON} + (1-p_{ON})N_{OFF}\right)\left(1 - \frac{N_{ON}+N_{OFF}}{K}\right) \quad \text{(S13)}$$

The p_{ON} and p_{OFF} are state change probabilities for cells turning ON and OFF respectively. The system of differential equations in Equation S13 is autonomous – the slopes do not depend upon t, so we can visualize the behaviors of the solutions using a vector field plot as shown in Figure S4.

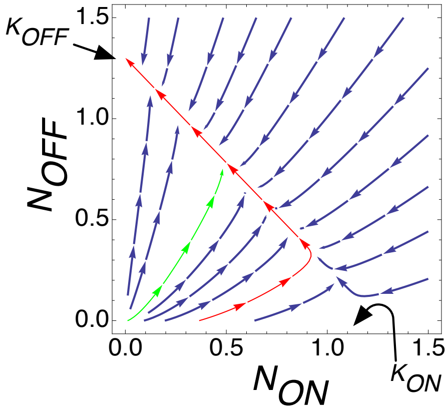

Figure S4: A vector field showing the direction of the slopes for the system of differential equations shown in S13. Units are normalized. As long as $K_{OFF} > K_{ON}$. Two special situations are shown: The green arrows show the trajectory of equal but small initial concentrations of cells growing at the same level until $N_{ON} + N_{OFF} = K_{ON}$. Subsequently the vector field shifts such that the N_{ON} population goes to zero and the N_{OFF} population approaches K_{OFF}. The red arrow denotes the situation when an OFF phenotype appears in a steady state culture of ON phenotypes.

The vector plot above is insensitive to the growth rate ρ, which can be readily seen since $\frac{d}{d\rho}\frac{dN}{dt}$ is independent of ρ. The key conclusion from the Figure S4 is that as long as $K_{OFF} > K_{ON}$, the only stable point in this system is $(0, K_{OFF})$, or when all the cells have switched OFF. The rate at which the system approaches $(0, K_{OFF})$ depends upon a host of factors, including relative values of K_{ON} ,K_{OFF} as well as the probabilities p_{ON}, p_{OFF} and the starting concentrations N_{ON_0} and N_{OFF_0}.

S2.2 Sensitivity analysis for the competitive growth model

We solved the numerical differential equations shown in Equation S13 using parameter estimates for ρ and K for the ON and OFF phenotypes obtained from Figure 5 in the main text. The model sensitivity was visualized for changes in dilution rate, changes in the stochastic switching probability as well as sensitivity to the initial ratio of ON and OFF phenotypes. The dependence of the dilution rate is shown in the main text (Figure 8). The following Figure S5 shows the sensitivity to switching probabilities and initial distribution of the phenotypes.

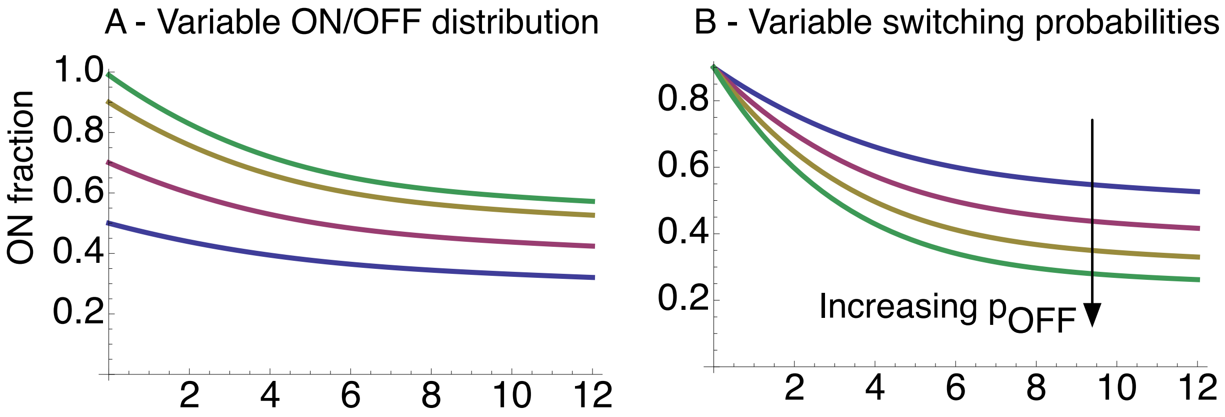

Figure S5: Numerical estimates of dependence of the ON fraction solely on A) the initial ON - OFF distribution, and B) variation in the probability of switching between the ON and OFF fractions. The model behaves as expected, i.e. the rate at which the ON population fraction decreases is directly proportional to the initial distribution of the ON population, and is indirectly proportional to the probability with which an ON phenotype will switch to OFF.

S3 Solving for the constants of the dynamical system

The experimental data for induction of the feedback circuit presented in Figure 5 of the main text can be fitted to Equation S1. Figure S6 shows the optical density measurements for the cells grown at the various inducer levels. Numerical derivatives were calculated for each of GFP expression curves. The derivatives were then fit to the

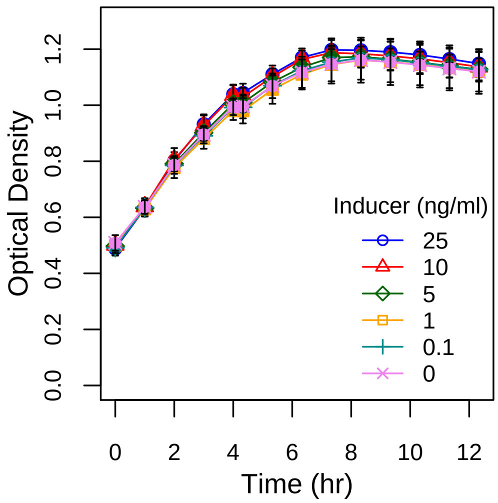

Figure S6: Optical density measurements for cultures grown under various inducer levels corresponding to Figure 5 in the main text. All cell cultures grow at the same rate.

nonlinear model shown in S1. The R® statistical programming environment was used to compute for the constants k_1 through k_4 for the model. A summary output of the results of the nonlinear regression is reproduced below:

```
Summary of the nonlinear regression analysis
Parameters:
    Estimate Std. Error t value Pr(>|t|)
k1 1.005e+01  1.848e+00   5.439 1.22e-06 ***
k2 3.482e-01  5.003e-02   6.958 4.06e-09 ***
k3 3.710e+01  2.481e+00  14.957  < 2e-16 ***
k4 1.032e+03  9.742e+01  10.597 5.36e-15 ***
---
Signif. codes:  0 *** 0.001 ** 0.01 * 0.05 . 0.1   1

Residual standard error: 76.3 on 56 degrees of freedom

Number of iterations to convergence: 14
```

```
Achieved convergence tolerance: 2.544e-06
```

Although technically g in the model refers to the expression level of $luxR\Delta$, we use the relative fluorescence units (RFU) of green fluorescent protein to fit the model. The inducer level is measured in ng/ml and time is measured in hours. Table S2 lists the units of the model for all the terms:

Term	Estimate	Std. Error	Unit
g	data		RFU (relativel fluorescence units)
t	data		hours (h)
s_0	data		ng/ml
k_1	10.05	1.85	RFU.ml/ng/h
k_2	0.348	0.05	h^{-1}
k_3	37.1	2.48	RFU$^{1/2}$.h$^{1/2}$
k_4	1032	97.4	RFU

Table S2: Units for the terms in Equation S1.

Corresponding to the fitted values of the constants k_1 through k_4 for given above, we can calculate the values of r and s for their dimensionless counterparts. The degradation constant $r = \frac{k_2 k_4}{k_3^2}$=(0.26±0.058) , and $s = \frac{k_1 s_0}{k_3^2}$=(0.0074±0.001)$\times s_0$. The error estimates on r and s were obtained using Monte Carlo simulation on the values of the constants k_1 through k_4.

The value of $r < 0.5$ indicates that the system should produce irreversible hysteresis, and for a induction level of $s_0 < 2.4$ ng/ml, the system will be bistable.

S4 Flow cytometry data for the dilution experiment

The dilution experiments were analyzed using flow cytometry to quantify the distribution of the cells in the ON-OFF state. The results of the flow cytometry are shown in Figure S7 below.

From Figure S7, the following phenomena are apparent:

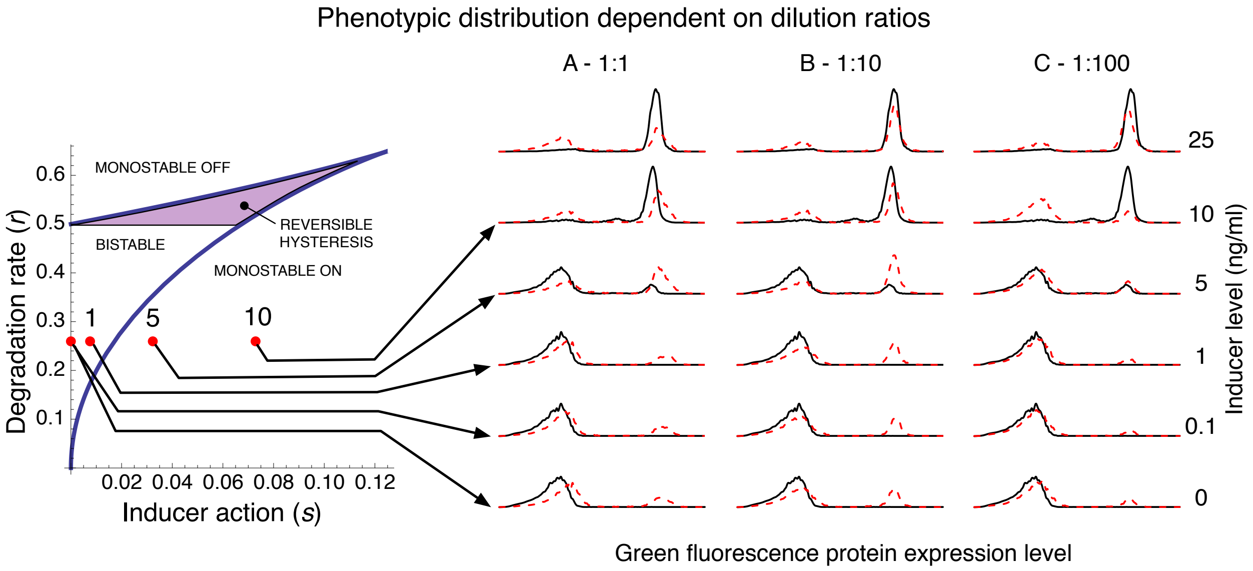

Figure S7: Flow cytometry data. Each subgraph is a histogram showing the distribution of cells expressing GFP. The two peaks correspond to cells showing the two different ON and OFF phenotypes. The three columns of subfigures each represent the three different dilution procedures. Each row corresponds to an induction level. The solid lines represent the induction of naïve, untreated cells at various inducer levels. The dotted lines represent the cells induced at 25 ng/ml and subsequently diluted to the various inducer levels.

1. For cells resuspended using a 1:1 dilution ratio at high induction levels (5, 10, and 25 ng/mL) the proportion of ON cells ranges from 47-59%, and at low induction levels (0, 0.1, and 1 ng/mL) the proportion of ON cells ranged from 21-23%.

2. For cells resuspended using a 1:10 dilution ratio at high induction levels the proportion of ON cells ranges from 58-70%, and at low induction levels the proportion of ON cells ranged from 28-33%.

3. For cells resuspended using a 1:100 dilution ratio at the highest induction level (25 ng/mL) the proportion of ON cells was 71%, and at all lower induction levels the proportion of ON cells ranged from 8-19%.

Thus, at higher dilution (1:100), the proportion of ON cells remaining seems to be smaller than at lower dilutions (1:10 / 1:1)

The following Figure S8 exemplifies this using flow cytometry data from groups A and B at an inducer concentration of 0.1 ng/mL. Group B (dashed line) shows a higher proportion of cells in the ON state, but the location of the mode for the ON subpopulation is lower than the mode of the group A ON subpopulation. In other words, the cells from group A are expressing at a slightly higher steady-state level than group B. The trend is similar for the other inducer concentrations.

Additionally, for each inducer concentration, the ON cells from dilution group C (1:100) express at a slightly lower steady-state level than ON cells from group B.

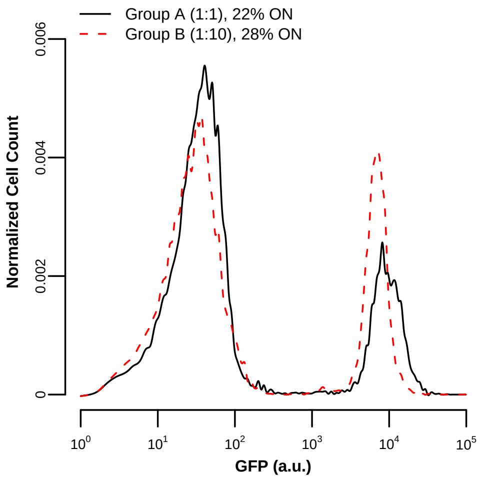

Figure S8: Flow cytometry histograms for cells from groups A and B at 0.1 ng/mL aTc. Cells from each dilution group exhibit hysteresis, but group B contains a higher proportion of ON cells than group A. However, the steady-state fluorescence level (mode) of the group A ON subpopulation is greater than the mode for group B. Thus, group A has a higher overall magnitude of expression as measured by relative fluorescence, but a lower proportion of ON cells, indicating that the dilution ratio used during resuspension may be affecting the steady-state expression level.
